# Mode of Neonatal Delivery Influences the Nutrient Composition of Human Milk: Results From a Multicenter European Cohort of Lactating Women

**DOI:** 10.3389/fnut.2022.834394

**Published:** 2022-04-06

**Authors:** Tinu M. Samuel, Frank Thielecke, Luca Lavalle, Cheng Chen, Paul Fogel, Francesca Giuffrida, Stephane Dubascoux, Cecilia Martínez-Costa, Kirsti Haaland, Giovanna Marchini, Massimo Agosti, Thameur Rakza, Maria Jose Costeira, Jean-Charles Picaud, Claude Billeaud, Sagar K. Thakkar

**Affiliations:** ^1^Nestlé Research, Société des Produits Nestlé S.A., Lausanne, Switzerland; ^2^Department of Health Promotion, Swiss Distance University of Applied Sciences, Regensdorf, Brig, Switzerland; ^3^Advestis, Paris, France; ^4^Hospital Clínico Universitario, University of Valencia, Valencia, Spain; ^5^Oslo University Hospital, Oslo, Norway; ^6^Karolinska University Hospital, Stockholm, Sweden; ^7^Ospedale del Ponte, Varese, Italy; ^8^Centre d’Investigation Clinique de Lille, Hôpital Jeanne de Flandre, Lille, France; ^9^Instituto de Investigação em Ciências da Vida e Saúde, Braga, Portugal; ^10^Hôpital de la Croix Rousse, Lyon, France; ^11^Hôpital des Enfants, CHU Pellegrin, Bordeaux, France; ^12^Nestlé Research, Société des Produits Nestlé S.A., Singapore, Singapore

**Keywords:** C-section = caesarean section, vaginal birth, mode of delivery, human milk, inflammation, longitudinal cohort

## Abstract

**Background:**

The effect of the mode of neonatal delivery (cesarean or vaginal) on the nutrient composition of human milk (HM) has rarely been studied. Given the increasing prevalence of cesarean section (C-section) globally, understanding the impact of C-section vs. vaginal delivery on the nutrient composition of HM is fundamental when HM is the preferred source of infant food during the first 4 postnatal months.

**Objective:**

This study aimed to evaluate the association between mode of delivery and nutrient composition of HM in the first 4 months of life.

**Design:**

Milk samples were obtained from 317 healthy lactating mothers as part of an exploratory analyses within a multicenter European longitudinal cohort (ATLAS cohort) to study the HM composition, and its potential association with the mode of delivery. We employed traditional mixed models to study individual nutrient associations adjusted for mother’s country, infant birth weight, parity, and gestational age, and complemented it, for the first time, with a multidimensional data analyses approach (non-negative tensor factorization, NTF) to examine holistically how patterns of multiple nutrients and changes over time are associated with the delivery mode.

**Results:**

Over the first 4 months, nutrient profiles in the milk of mothers who delivered vaginally (*n* = 237) showed significantly higher levels of palmitoleic acid (16:1n-7), stearic acid (18:0), oleic acid (18:1n-9), arachidic acid (20:0), alpha-linolenic acid (18:3n-3), eicosapentaenoic acid (20:5n-3), docosahexenoic acid (22:6n-3), erucic acid (22:1n-9), monounsaturated fatty acids (MUFA)%, calcium, and phosphorus, whereas the ratios of arachidonic acid/docosahexaenoic acid (ARA/DHA) and n-6/n-3, as well as polyunsaturated fatty acids (PUFA)% were higher in milk from women who had C-sections, in the unadjusted analyses (*p* < 0.05 for all), but did not retain significance when adjusted for confounders in the mixed models. Using a complementary multidimension data analyses approach (NTF), we show few similar patterns wherein a group of mothers with a high density of C-sections showed increased values for PUFA%, n-6/n-3, and ARA/DHA ratios, but decreased values of MUFA%, 20:1n-9, iodine, and fucosyl-sialyl-lacto-N-tetraose 2 during the first 4 months of lactation.

**Conclusion:**

Our data provide preliminary insights on differences in concentrations of several HM nutrients (predominantly fatty acids) among women who delivered *via* C-section. Although these effects tend to disappear after adjustment for confounders, given the similar patterns observed using two different data analytical approaches, these preliminary findings warrant further confirmation and additional insight on the biological and clinical effects related to such differences early in life.

## Introduction

Cesarean section (C-section), as a medical intervention, has been increasingly overused in several parts of the world, with a doubling in the proportion between 2000 and 2015 (an increase from 12.1 to 21.1%) and an estimated 6.2 million non-medically indicated C-sections being performed each year (1–4). In Europe specifically, this has reached a rate of approximately 27% (2). Compared to vaginal birth, C-sections confers increased health risks to the mother, such as increased risk of maternal mortality, severe acute morbidity, and higher risk for adverse outcomes in subsequent pregnancy (e.g., hysterectomy, abnormal placentation, uterine rupture, stillbirth, and preterm birth) (5), in addition to psychological effects (e.g., less satisfactory childbirth experience, more likely to experience postnatal depression, anxiety, lower self-esteem, difficulties in the mother–infant relationship, and in breast-feeding) (6). Infants born via C-section, compared with those born vaginally, are at an increased risk of altered immune development, allergy, atopy, asthma, and reduced diversity of the intestinal gut microbiome (5). Recent evidence also suggests that C-section may be associated with the neurodevelopmental outcomes such as a shift in brain development, at least during early infancy (e.g., significantly lower white matter development in widespread brain regions and significantly lower functional connectivity in the brain default mode network) (7). These have been postulated to be mediated *via* biological mechanisms, such as inadequate transfer of the maternal microbiome to infants born *via* C-section leading to altered immunological development, reduced intrapartum exposure to physical forces of labor and stress hormones that may bypass important signals in the infant for development of the hypothalamic–pituitary–adrenal axis, maturation of the immune system, lung and organ maturation, and neurogenesis, and possibly *via* differences in epigenetic modification of gene expression (5).

Considering the impact of C-section on several aspects of maternal and child health, it would be important to understand how the mode of delivery (whether vaginal or C-section) influences the composition of human milk (HM) and to assess whether such differences in HM are related to later health outcomes among C-section born infants, at least when HM is the primary source of food. HM, while being the optimum source of nutrition for infants, provides a multitude of nutrients from birth till weaning. The composition and roles of key nutrients are reviewed by many, and we recommend Ballard et al. (8) where information is summarized. While the mode of delivery has been associated with variations in microbiome or microbiota composition (9–11), very few studies have reported the effect of mode of delivery on the composition of HM. In two multicenter studies, differences were observed in (HM) microbiome and polyamines based on the mode of delivery, and these differences were further enhanced depending on the geography (12, 13).

Currently, the influence of mode of delivery on the nutrient composition of milk remains unclear. Therefore, the present study aimed to identify potential associations between the mode of delivery and the nutrient profile of HM in a multicenter longitudinal study in European lactating mothers.

## Materials and Methods

### Study Population and Design

ATLAS is a multicenter, longitudinal, observational, cohort study designed to characterize HM and its association with maternal and infant parameters (14). Enrollment was performed at multiple sites in 7 European countries, including Spain, France, Italy, Norway, Portugal, Romania, and Sweden. The total duration of participation was 4 months after infant birth. HM collection was standardized across the sites, and timing of collection was fixed at 1100h ± 2 h. Trained and certified research nurses and assistants collected all data from the years 2012–2014. All data captured were directly entered into a secured web-based database (Medidata Rave edc 5.6.4). Participant inclusion and exclusion criteria are shown in Table 1. The procedures followed were according to the ethical standards of the respective local ethical committees in each country. We acknowledge that there has been a decrease in the number of mothers and infants over the first 4 months of lactation. This decrease was observed in both the groups and is anticipated in most infancy studies with longitudinal data collection and intense milk sampling.

**TABLE 1:** Participant inclusion and exclusion criteria.

**Inclusion criteria**
Between 18 and 40 of age at the time of enrollment
BMI before pregnancy between 19 and 29, inclusive
Having decided to exclusively breast-feed from birth up to 4 months of age
Signed the informed consent form
**Exclusion criteria**
Participation in another clinical study during the 4 weeks prior to the beginning of this study
Medical condition which prevented breast-feeding or collection of human milk samples or for which breast-feeding is not indicated
Diseases/medical conditions such as diabetes, heart problems, abnormal conditions of pregnancy (e.g., hypertension), anorexia, bulimia, and celiac disease
Medications indicated for the treatment of any metabolic or cardiovascular disease
Non-compliance with the study procedures
Mothers given birth to twins

### Collection of Human Milk Samples

The HM sampling was standardized for all participants. HM as well as multiple maternal and infant parameters were collected postpartum at 6 visits (V) (V1, 0–3 days; V2, 17 ± 3 days; V3, 30 ± 3 days; V4, 60 ± 5 days; V5, 90 ± 5 days; V6, 120 ± 5 days). Milk was collected at 1100h ± 0200h using an electric breast pump. The side of the breast selected by the mother was kept the same for the entire study and mothers were requested to empty the breast in the previous feed or pumping session. Single full breast was sampled and an aliquot of 10–40 mL HM for each time point was reserved for biochemical characterization. Colostrum, or the first time point collected, was limited to 5–10 mL. The remainder of the milk was returned to the mother to feed the infant at a later point, if required. Each sample was transferred to freezing tubes, stored at −18°C in the home freezer, transferred to the hospital (storage at −80°C), and then shipped to the analysis center where it was stored at −80°C until analysis. The frozen HM samples were thawed once for aliquoting into 15 individual small volume fractions (min 0.2 mL to max 2 mL) in separate polypropylene Eppendorf tubes dedicated to the different analyses. The aliquoting approach was implemented to avoid repeated thawing–freezing cycles and to adapt the required volumes to the specific needs of the individual analytical methods.

### Analytics of Nutrients in Human Milk

Total lipids in HM were analyzed by mid-infrared spectroscopy (HMA, Miris AB, Uppsala, Sweden) as previously reported (15). Total protein content in HM was measured using the colorimetric bicinchoninic acid (BCA) method according to the manufacturer’s protocol of BCA assay kit (Thermo Fisher Scientific, Waltham, MA, United States). The four major HM proteins: alpha-lactalbumin, lactoferrin, serum albumin, and caseins, were quantified using a LabChip system (16).

#### Fatty Acid Analysis

Fatty acids were quantified by gas chromatography coupled with a flame ionization detector (17). Briefly, after the addition of 300 μL of internal standard fatty acid methyl ester 21:0 and 100 μL of internal standard triglyceride 13:0, 2 mL of methanol, 2 mL of methanol/HCL (3N) and 1 mL of *n*-hexane were added. The tubes were heated at 100°C for 60 min. After cooling down the tubes to room temperature, 2 mL of water was added. After centrifugation at 1,200 × *g* for 5 min, the upper phase (hexane) was injected into a gas chromatograph equipped with a CP-SIL 88 column (Agilent J&W GC columns, Part# CP7489).

#### Mineral Analysis

Sodium (Na), magnesium (Mg), phosphorous (P), potassium (K), calcium (Ca), manganese (Mn), iron (Fe), copper (Cu), zinc (Zn), and selenium (Se) were quantified by inductively coupled plasma tandem mass spectrometry (18). Briefly, 700 μL of HM aliquots were mineralized by microwave after the addition of HNO_3_/H_2_O_2_. Then, after additional dilution and addition of internal standards, quantification was realized by inductively coupled plasma-mass spectrometry (ICP-MS) using helium collision mode for Na, Mg, P, K, Ca, Mn, Fe, Cu, and Zn and CH_4_ reaction mode for Se.

For iodine (I), 1 mL of HM was treated with 1 mL tetramethylammonium hydroxide (25%) and 4.5 mL of MQ water in a drying oven at 90°C for 3 h. Sample solutions were diluted to 15 mL and centrifuged at 1,730 × *g* for 15 min. The supernatant was analyzed by ICP-MS in normal mode using Germanium as the internal standard.

#### Human Milk Oligosaccharide Quantification

*Human milk oligosaccharide*s were quantified by liquid chromatography with fluorescence detection after labeling with 2-aminobenzamide (14). HMOs were quantified against genuine standards, when available, with known purity. All other HMOs were quantified against maltotriose with known purity, assuming equimolar response factors.

### Statistical Analysis

A wide range of nutrients in HM have been analyzed, of which we focused on 90 for this study. Values below the level of quantification for a given parameter were replaced by half of the corresponding level of quantification.

First, a targeted analysis was conducted to test whether each individual’s milk nutrient varied according to the delivery mode. Furthermore, while targeted analysis looks at each milk nutrient one by one, non-negative tensor factorization (NTF) was used to examine the temporal evolution by considering nutrients clustered together. NTF identifies clusters of nutrients and clusters of subjects who show common temporal evolution patterns, and the clustering of subjects was shown to be associated with the mode of delivery.

To compare levels of the milk components between delivery modes at each visit, two-sided Mann–Whitney *U* tests were performed. Additionally, to account for confounders, a targeted approach (mixed model) was fitted to each of these 90 components. Such a model was fitted on the log-transformed^1^ data to achieve approximate normality of the residuals. Individuals (pairs of a mother and her child) were considered as random effects and the following variables were considered as main fixed effects in the mixed model: visit, delivery mode, and the interaction between the latter two. In addition, mother’s country, infant’s weight at V0 (birth weight), parity, and gestational age were considered as potential confounders and were also added as fixed effects. Due to log transformation, group differences between delivery modes were computed per visit and were expressed as a ratio of geometric means between the vaginal group and the C-section group. Hence, a model-based estimate higher than 1 means that the estimation (geometric mean) is higher in the vaginal group than in the C-section group, while an estimate below 1 means that levels of the corresponding parameter are higher in the C-section group. A value of *p* < 0.05 was considered statistically significant. Owing to the data-driven and exploratory nature of the analyses, we did not control for the multiplicity.

#### Non-negative Tensor Factorization

**FIGURE 1:**
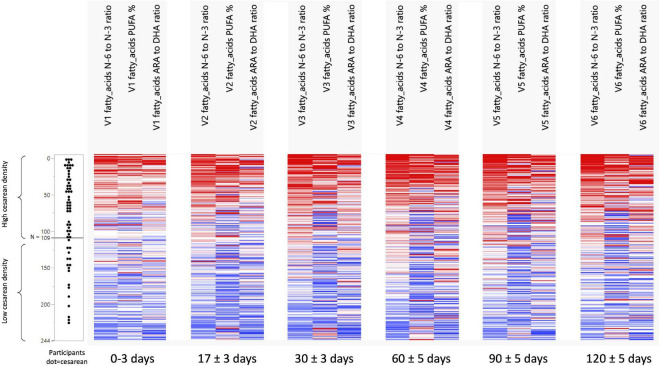
Development of n-6/n-3 ratio, PUFA%, and the ARA/DHA ratio across visits and per C-section density grouping. N 0 to N 109 = the group with a high-density C-section group. N 110 to N 244 = the group with a low-density C-Section group.

**FIGURE 2:**
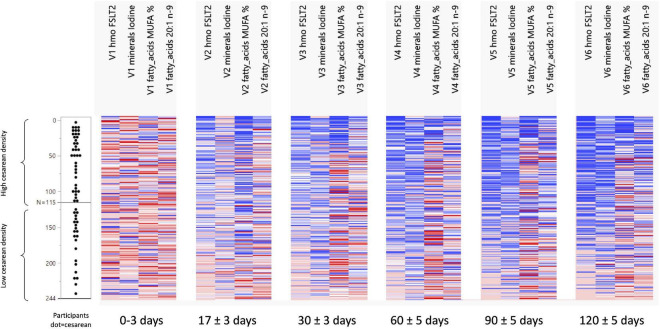
Development of FSLT2, Iodine, MUFA%, and 20:1n-9 ratio across visits and per C-section density grouping. N 0 to N 115 = the group with a high-density C-section group. N 116 to N 244 = the group with a low-density C-Section group.

Non-negative tensor factorization is a non-supervised approach dedicated to the analysis of longitudinal datasets (19). Specifically, NTF estimates several factors along the three dimensions of the data space: participant × visit × HM component. Each NTF factor represents a particular trend (e.g., increase/decrease). Participant and parameter loadings along each NTF factor reflect the level of similarity for a given participant and parameter with one trend or another. NTF factor loadings are subsequently used to build bi-clusters of participants and milk parameters sharing similar trends. To guide the NTF analysis for greater focus on the mode of delivery, pre-selected nutrients were included. Repeated ANOVA with the delivery mode as a main effect on 62 milk nutrients (log-transformed), using JMP version 14.2 (SAS Institute), was conducted to pre-select those nutrients. Participants with missing time points or data were excluded in the repeated ANOVA. In this way, only nutrients were analyzed by NTF that showed some degree of association with the mode of delivery. NTF results are presented as heatmaps (Figures 1, 2), with rows corresponding to mothers and columns corresponding to human milk components. We found that the top rows grouped mothers with a high proportion of C-section deliveries (later referred to as high C-section density), while the bottom rows had a lower proportion of C-section deliveries (later referred to as low C-section density). Therefore, we searched for a cutoff row to divide the subjects into high and low C-section density groups. To do this, we first assigned all subjects to the high-density group, minus the 20 subjects at the bottom who were assigned to the low-density group. We then performed a repeated ANOVA on the sum of metabolites (with the visit, the high- vs. low-density group, and the interaction of the latter two as independent variables), removed the bottommost subject from the high-density group, and repeated the procedure until the significance was greater than 0.05.

## Results

**FIGURE 3:**
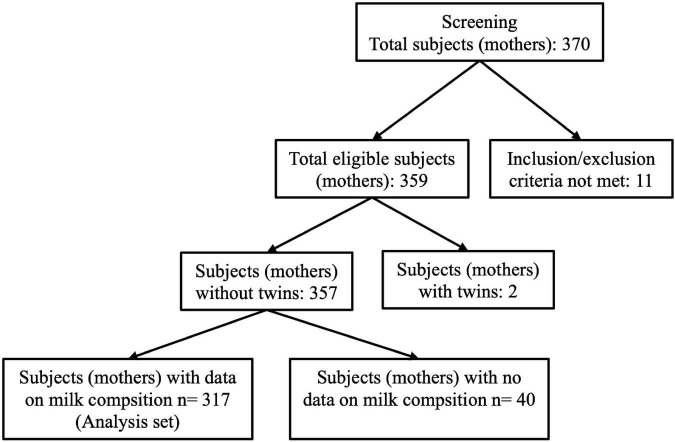
Flow chart on the cohort analysis.

A total of 370 participants from 7 European countries including Spain (1 center), France (3 centers), Italy (1 center), Norway (1 center), Portugal (3 centers), Romania (2 centers), and Sweden (2 centers) were enrolled for this study and included in the dataset. Participants were counted as pairs of mothers and infants. The current analysis was performed on 317 participants (pairs) after removal of pairs who did not satisfy the inclusion–exclusion criteria, pairs with twins, and with incomplete information on HM composition (Figure 3).

Two hundred thirty-seven mothers delivered vaginally, whereas 80 had a C-section. Mothers’ height was significantly lower in the C-section group (Table 2). France and Portugal represented the biggest groups with 88 and 100 mothers, respectively. Vaginal birth was the main mode of delivery for all countries, except Romania, where 34 vs. 10 mothers delivered by C-section. For 239 mothers it was their first delivery while for 78 mothers it was their second or subsequent delivery. Over the first 4 visits, the infant’s head circumference was significantly smaller in the C-section group compared with infants delivered vaginally. This leveled off and no significant differences were observed at V5 and V6 days postpartum. While infant’s crown-heel lengths did not differ much in absolute terms, significantly smaller lengths were noted for V2 to V4. The differences between C-section and vaginal delivery were more pronounced for infant weight, which was consistently lower in the C-section group across all visits and was statistically significant for V0 to V4 (*p* < 0.05).

**TABLE 2:** Maternal and infant characteristics.

		C-section (Tot. = 80)			Vaginal (Tot. = 237)			
	Visits	*n*	*Median*	[Q1,Q3] or %	*n*	*Median*	[Q1,Q3] or%	*P*-Value
**Mother’s age**								
		80	32	[28, 34]	237	31	[28, 34]	0.45
Maternal pre-pregnancy BMI		80	22.2	[22.6, 25.2]	237	22.1	[22.6, 25.2]	0.59
**Mother’s BMI**								
	Child’s birth	78	28.1	[24.5, 31.1]	221	27.4	[25.0, 29.7]	0.43
	0–3 days	79	26.6	[23.8, 29.9]	227	25.5	[23.6, 28.3]	0.1
	17 ± 3 days	73	25.4	[22.4, 27.3]	218	24.4	[22.4, 26.7]	0.22
	30 ± 3 days	66	25.0	[21.9, 27.2]	200	24.1	[22.1, 26.4]	0.33
	60 ± 5 days	60	24.9	[22.1, 26.9]	186	23.9	[21.7, 26.2]	0.25
	90 ± 5 days	57	24.5	[22.2, 26.9]	176	23.7	[21.5, 26.0]	0.11
	120 ± 5 days	56	24.5	[21.9, 27.2]	167	23.3	[21.3, 25.5]	0.1
**Country**								
	Spain	3		3.80%	10		4.20%	< 0.01
	France	10		12.50%	78		32.90%	
	Italy	1		1.20%	19		8%	
	Norway	0		0%	9		3.80%	
	Portugal	28		35%	72		30.40%	
	Romania	34		42.50%	10		4.20%	
	Sweden	4		5%	39		16.50%	
**Parity**								
	Primiparous	68		85%	171		72.20%	0.03
	Multiparous	12		15%	66		27.80%	
**Gestational age**								
		80	39	[38.0, 39.0]	237	40	[39, 40]	< 0.01
**Infant’s head circumference**								
	Child’s birth	79	34.0	[33.0, 35.0]	237	34.8	[34.0, 35.5]	< 0.01
	0–3 days	78	34.0	[33.0, 35.0]	235	34.6	[34.0, 35.5]	0.01
	17 ± 3 days	73	35.4	[34.5, 36.6]	219	36.3	[35.5, 37.0]	< 0.01
	30 ± 3 days	66	37.0	[36.0, 38.0]	201	37.5	[36.5, 38.5]	< 0.01
	60 ± 5 days	60	38.9	[38.0, 39.5]	186	39.2	[38.3, 40.0]	0.02
	90 ± 5 days	57	40.5	[40.0, 41.0]	176	40.7	[39.5, 41.5]	0.49
	120 ± 5 days	56	41.5	[41.0, 42.2]	167	41.5	[41.0, 42.5]	0.61
**Infant’s length z-score**								
	Child’s birth	80	0.13	[−0.73, 0.74]	236	0.33	[−0.35, 0.99]	0.24
	0−3 days	79	–0.08	[−0.90, 0.40]	234	0.09	[−0.53, 0.77]	0.14
	17 ± 3 days	73	–0.49	[−1.14, 0.11]	219	0.00	[−0.67, 0.71]	< 0.01
	30 ± 3 days	66	–0.47	[−1.19, 0.12]	200	0.04	[−0.81, 0.71]	< 0.01
	60 ± 5 days	59	–0.50	[−1.12, 0.20]	186	–0.02	[−0.74, 0.66]	< 0.01
	90 ± 5 days	56	–0.24	[−0.93, 0.26]	176	–0.05	[−0.67, 0.67]	0.04
	120 ± 5 days	56	−0−−0.1	[−1.05, 0.49]	168	–0.06	[−0.68, 0.66]	0.27
**Infant’s weight z-score**								
	Child’s birth	80	–0.22	[−0.88, 0.41]	237	0.19	[−0.39, 0.87]	< 0.01
	0−3 days	79	–0.55	[−1.18, 0.04]	235	–0.13	[−0.78, 0.48]	< 0.01
	17 ± 3 days	73	–0.66	[−1.24, 0.03]	219	0.03	[−0.62, 0.53]	< 0.01
	30 ± 3 days	66	–0.61	[−1.02, 0.05]	201	0.07	[−0.65, 0.59]	< 0.01
	60 ± 5 days	60	–0.61	[−1.15, -0.02]	186	–0.11	[−0.77, 0.47]	< 0.01
	90 ± 5 days	57	–0.52	[−1.11, 0.35]	176	–0.15	[−0.79, 0.45]	0.06
	120 ± 5 days	56	–0.52	[−1.04, 0.30]	168	–0.17	[−0.86, 0.42]	0.13
**Infant’s sex**								
	Male	44		55%	127		53.60%	0.93
	Female	36		45%	110		46.40%	

*Sample size, median, 1st quartile (Q1 = 25th percentile), 3rd quartile (Q3 = 75th percentile), and p-value (Mann- Whitney U test) for continuous characteristics. For categorical characteristics, count, percentage, and p-value (Pearson’s chi-squared test).*

**FIGURE 4:**
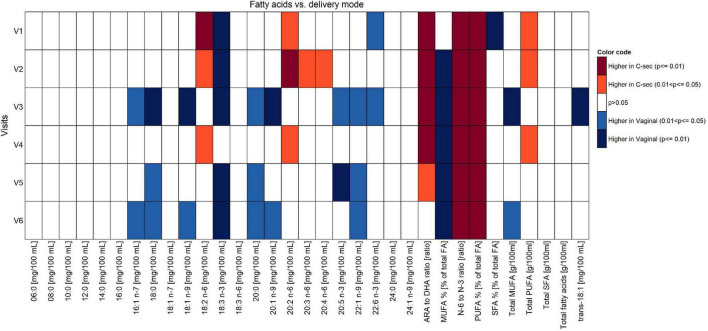
Comparison of fatty acid content between vaginal delivery and C-section per visit (Mann-Whitney test *U* test unadjusted for multiple comparisons).

A modified test weighing method was employed to determine the HM intake in a subset of the infants, as reported in detail in our previous publication (18). We observed a higher intake in the infants’ daily intake of HM over the first 4 months of lactation in the vaginal group as compared to the C-section group (about 78–91 g higher when averaging mean or median differences across study visits), although the individual differences from Mann–Whitney *U* tests were statistically not significant (Supplementary Figure 1). No differences between vaginal birth and C-section were found with respect to total protein, lactose, and lipid content. However, the effects of mode of delivery on fatty acids composition across V1 to V6 showed significantly higher concentrations of palmitoleic acid (16:1n-7), stearic acid (18:0), oleic acid (18:1n-9), arachidic acid (20:0), erucic acid (22:1n-9), alpha-linolenic acid (18:3n-3), eicosapentaenoic acid (20:5n-3), docosahexenoic acid (22:6n-3), and monounsaturated fatty acids (MUFA)% in the milk of women with vaginal delivery (*p* < 0.05; Figure 4), but did not retain significance when adjusted for confounders in the mixed models.

Focusing on V1 only, concentrations of 18:3n-3 and 22:6n-3, as well as SFA% were significantly higher in women with vaginal delivery (*p* < 0.05), whereas concentrations of 18:2n-6, 20:2n-6, ARA/DHA, n-6/n-3, polyunsaturated fatty acids (PUFA)%, and total PUFA were significantly higher in the C-section group (*p* < 0.05). See Supplementary Table 1 for absolute amounts of milk components.

**FIGURE 5:**
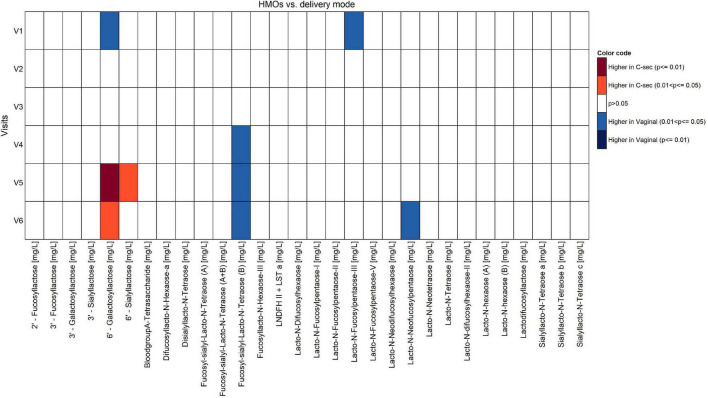
Comparison of human milk oligosaccharides between vaginal delivery and C-section per visit (Mann-Whitney *U* test unadjusted for multiple comparisons).

**FIGURE 6:**
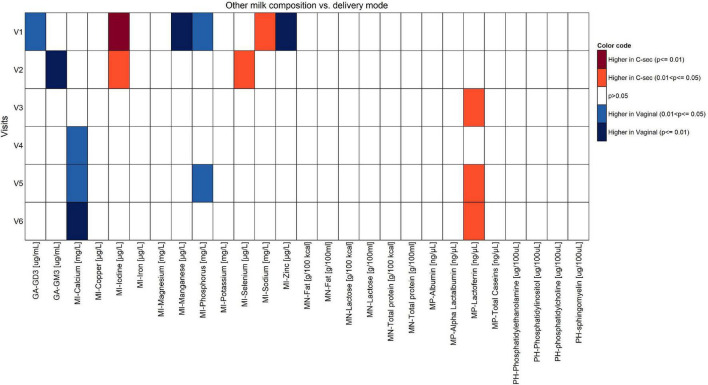
Comparison of components of human milk other than fatty acids between vaginal delivery and C-section per visit (Mann-Whitney *U* test unadjusted for multiple comparisons).

The comparison of the effects of vaginal delivery vs. C-section on the constituents of HM other than fatty acids is shown in Figures 5, 6. Concentrations of calcium and phosphorous were significantly higher in women giving birth vaginally (*p* < 0.05). For calcium, this was observed particularly in the second half of the lactation period V4–V6. Levels of leptin and lactoferrin were significantly higher in women with C-section across almost all visits (*p* < 0.05).

At V1, the levels of ganglioside GD3, manganese, phosphorous, and zinc were significantly higher in the vaginal delivery group (*p* < 0.05), whereas iodine and sodium were significantly higher in the C-section group (*p* < 0.05). Once confounders such as mother’s country, infant’s weight at V0 (child’s birth), parity, gestational age, and visit were included in the model, no statistical differences were observed (data not shown).

Observed trends and statistical differences from the non-parametric tests (Figures 4–6) were not confirmed in the mixed effect model results for most of the human milk components. However, for most of these, the mixed-model-based estimate and corresponding confidence intervals tend to agree with non-parametric results in the comparison between delivery modes, except for the components such as fatty acids 18:2 n-6 (V1 and V3), 18:3 n-3 (V1, V2, V4, V5, and V6), 20:2 n-6 (V3), 20:4 n-6 (V2), 20:5 n-3 (V2), ARA to DHA ratio (V1, V2, V3, V5, and V6), N-6 to N-3 ratio (V2, V4, and V6), PUFA% (V1 and V2), and total PUFA (V2) at selected few visits mentioned in the parentheses of each nutrient.

### Differences Human Milk Nutrients Between Groups With High vs. Low Density of C-Section

The NTF approach was used to identify groups of nutrients and groups of participants that showed similar temporal patterns. It was used on 244 participants with 17 pre-selected nutrients. Two groups of nutrients were identified: one with 3 nutrients (Figure 1) and one with 4 (Figure 2). Two groups of participants were identified: one was characterized as high-density C-section (i.e., a high proportion of C-section deliveries) and the other as low-density C-section (i.e., a low proportion of C-section deliveries). The density of C-section is represented as dots just right next to the subject index, and we see that there are many more dots in one group than the other.

Figure 1 shows that in the high-density C-section group (109 participants), the n-6/n-3 ratio, PUFA%, and the ARA/DHA ratio increased from V1 to V6 (illustrated by the higher density of red color), whereas no clear pattern emerged for the low-density C-section group (135 participants). The opposite pattern was found for the HMO fucosyl-sialyl-lacto-N-tetraose 2 (FSLT2), iodine, MUFA%, and 20:1n-9 (Figure 2). These parameters decreased over time in the high-density C-section group (115 participants). A similar trend was observed in the low-density group (129 participants).

Overall, the NTF analysis tends to support the results of the Mann-Whitney *U* test (unadjusted analysis), specifically for the ratios of n-6/n-3 and ARA/DHA, PUFA%, and iodine. More importantly, the NTF proved to be a tool to follow changes in the nutrient composition of HM across lactation. For example, iodine was significantly higher in the C-section group at V1 and V2. NTF additionally showed that iodine was higher in the high-density C-section group across V1 to V6.

## Discussion

For the first time, and as shown in a multicenter European cohort, we observed that concentrations of certain fatty acids and specific minerals in HM are impacted by the mode of delivery, when applying unadjusted analysis. Confounder-adjusted analysis did not result in statistically significant differences. Furthermore, while using the multidimensional data analyses approach that aims to discover patterns of nutrients over time specific to delivery mode and when comparing high vs. low density C-section groups across the first 4 months of lactation, the high-density group was associated with increased ratios of n-6/n-3 and ARA/DHA, increased PUFA%, and also decreased levels of FSLT2, iodine, MUFA%, and 20:1n-9.

In the current understanding of the influence of mode of delivery on HM composition, the HM microbiome has been researched the most. Transport of intestinal bacteria or ingested probiotics to HM has previously been reported from clinical studies, where C-section was shown to have an independent effect on microbiota of HM. The effects were more profound on the milk microbiota composition as compared to intrapartum antibiotic exposure (20). However, the clinical implications for an altered HM microbiota are not fully understood, but evidence suggests that HM microbiota supports the appropriate maturation of the infants immune system (21). While the impact of mode of delivery on the HM microbiome is still under debate (10, 22–24), not much attention has been paid to HM nutrients, such as certain fats, that may also have an impact on later programming of health and disease (13, 25).

In contrast to others, we found no effect of C-section on total lipid or carbohydrate content. For example, a multivariate logistic regression analysis of 478 HM samples showed a positive association between lipid content in HM and C-section (OR = 2.47, *p* < 0.001), and a higher carbohydrate content was associated with vaginal delivery (OR = 0.50, *p* = 0.005) (26). The association of higher carbohydrate and protein content with vaginal delivery was also reported in colostrum and mature milk after pre-term delivery (27). Concordantly, a study evaluating the effect of mode of delivery on macronutrient content in colostral milk of 204 lactating mothers found protein levels in colostral milk in the C-section group significantly lower when compared with the vaginal delivery group (median 2.4 (range 0.3–6.4) g/dl versus 3 (0.5–6.3) g/dl, respectively; *p* = 0.036) (28). The same study reported no differences between groups in energy levels, lipid, or carbohydrate levels, which supports our findings. When applying linear regression analysis, (28) revealed that, next to C-section delivery, maternal age was also independently associated with lower protein content in colostrum. It may be hypothesized that stress-related hormone release induced by labor pain and uterine contractions might account for the alterations in the protein composition of HM to facilitate the optimal development of important physiologic functions in newborns (28). Differences across studies may be attributed to the population or regional differences, differences in dietary habits and environmental factors, differences in methodology for analyses of HM components, stage of lactation when HM was analyzed, confounding factors that were adjusted for, or even differences in sample size.

Inflammation is a response of the body’s immune system. N-3 and n-6 fatty acids, especially ARA, EPA, and DHA, are involved in the inflammatory response with ARA and the increased ratio of n-6/n-3, favoring overall a pro-inflammatory state. It is noteworthy that our study revealed higher ratios of ARA/DHA and n-6/n-3 PUFAs, as well as PUFA% in the C-section group in the unadjusted analyses. The latter is likely caused by the increased content of n-6 fatty acids. It has been reported that high levels of total n-6 PUFAs in HM are associated with an increased risk for asthma-like symptoms, whereas high levels of total n-3 PUFAs decreased the risk of atopy (31). Clinical evidence supports that n-3 PUFA supplementation, starting during pregnancy and continuing through 3.5 months of lactation, is associated with reduced severity of the allergic phenotype (32). Equally, a recent meta-analysis has shown that C-section increased the risk of childhood asthma (33). Putting these two together, one could speculate that the specific pattern of fatty acids observed in the milk of women who delivered *via* C-section in our study may have short- and long-term consequences for the child, although given the exploratory nature of our study, these observations need additional confirmation in future studies.

Furthermore, our finding that zinc and ganglioside (GD3) are lower in colostral milk of the C-section group may add to the increased inflammatory state in HM of those mothers. Although we do not have the data showing that women in our cohort had inadequate zinc intakes or were deficient in zinc, it has been reported that zinc deficiency can contribute to elevated inflammatory responses (34). Gangliosides, on the other hand, are involved in multiple aspects of the mucosal immune system (35). The inhibitory role of GD3 on dendritic cell functionalities suggests an additional role for GD3 in promoting tolerance against non-aggressive antigens, as GD3 levels in milk were reported to be higher in the colostrum (36). Higher values of GD3 in colostral milk after vaginal delivery could positively influence infants’ gut maturation over the course of lactation (37). However, a cross-sectional study in 540 HM samples assessing the role of C-section on immune factors found very little impact (16). In the same study, immunoglobulins (IgA, IgM, and IgG) and transforming growth factor (TGF) β1 and β2 were not significantly different when compared with vaginal delivery. Although we did not test for antioxidative status in our study, evidence suggests that C-section is associated with increased oxidative stress in colostrum when compared with vaginal delivery (38), which, overall, would support that C-section may result in a HM nutrient composition that favors a pro-inflammatory state (predominantly n-6 PUFA). The reason why these effects could not be retained when we adjusted for confounders including mother’s country, infant’s weight at birth, parity, gestational age, and visit maybe the lack of power to detect such differences, given the exploratory nature of our analyses. Nevertheless, given that we observe similar patterns when complemented with an untargeted approach, our findings merit further investigation. Beyond the preliminary step of hypothesis generation from this exploratory analysis, which is critical, we recommend that future confirmatory studies should perform in-depth analyses answering a pre-defined question supported by an adequate sample size that is powered to show such differences.

The content of calcium and phosphorous in HM has been researched primarily in the context of pre-term vs. term HM (39), but not compared by mode of delivery. In our study calcium and phosphorous in HM were significantly higher in women with vaginal delivery (*p* < 0.05). Furthermore, we found iodine increased at V1 after C-section (*p* < 0.05), which was also reported in a study with 444 lactating women (40). The authors highlighted the fact that this iodine increase in C-section groups could be due to the thyroxine (iodine-containing molecule) or the protein-bound iodine released after C-section and also to the use of povidone–iodine (also largely known as betadine^®^) as an antiseptic widely used for skin disinfection during C-section surgery. However, most centers in our study did not use betadine, with some exceptions. This study also reported that overall concentrations of most minerals were higher in the first 1 or 2 months of lactation, and then decreased with time. Interestingly, lactating women with C-section also had higher concentrations of iodine in the transitional milk (349.9 μg/kg) compared with vaginal delivery (237.5 μg/kg, *p* < 0.001). However, in the aforementioned study, maternal dietary intakes of minerals in the previous 6 months, maternal age, and maternal BMI did not show significant correlations with milk mineral concentrations.

While the targeted analysis looked at each milk nutrient one by one, the NTF analysis examined all nutrients together. By identifying clusters of nutrients, which evolve in a similar temporal pattern, NTF sheds more light by showing a broader picture of the potential association of C-section on changes in milk nutrients. In the NTF analysis, when comparing the high vs. low density C-section groups across the first 4 months of lactation, the high-density group was associated with increased ratios of n-6/n-3 and ARA/DHA, as well as increased PUFA%, but decreased levels of FSLT2, iodine, MUFA%, and 20:1n-9. The pattern of increased inflammatory fatty acids longitudinally supports our findings at the individual visits. Furthermore, applying NTF revealed a pattern of decreasing FSLT2 content across 4 months of lactation in the high-density C-section group. A separate analysis on the impact of delivery mode on the HMO composition has been published recently (14).

Many of our observations were in early lactation/colostrum and some of changes were more prominent in HM in the colostrum samples. We can only speculate on potential reasons for this finding. C-section was previously related with significantly lower levels of stress-associated hormone release when compared with vaginal delivery (41). Alterations in the physiological and hormonal signaling differ during C-section compared with vaginal delivery, which has been shown for milk microbiota (13). In addition, vaginal birth influences opioid galactopoiesis by increasing beta-endorphin content in colostral milk twofold, which in turn suggests that labor-related stress during parturition supports the newborn to better overcome vaginal delivery (42). Childbirth is triggered by several paracrine and autocrine events and fetal hormonal changes (43). Whether such hormonal changes may influence milk nutrients needs further investigation. In our study, we observed a higher daily milk intake in the Vaginal group as compared to the C-section group across all study visits. Particularly at V1 and V2, infants in the C-section group consumed less HM compared with infants delivered vaginally, possibly due to slow recovery after C-section, but this was not statistically significant. C-section has been shown to delay lactogenesis (44). Although the C-section group was under-represented in our analysis, it supports a report where the volume of HM transferred to infants born by C-section was significantly less during 2–5 days postpartum than that transferred to infants born by vaginal delivery (*p* < 0.05) (45). The first 5 days postpartum up to V4 may contribute to our finding that infant’s weight was higher in infants born vaginally compared with those born by C-section. We acknowledge that maternal height is related to both the mode of delivery and the infant weight, as suggested in previous literature (29, 30). Therefore, the lower weights observed among the C-section born infants may be a consequence of shorter maternal height or the delivery mode itself.

Ours is the first European longitudinal cohort with the characterization of HM nutrients, collected, and analyzed in a standardized way. It is also the first analysis of the impact of mode of delivery on HM nutrients in a longitudinal manner. Furthermore, the targeted statistical analyses were complemented by an untargeted approach (NTF). Many data are naturally organized in a multidimensional structure. NTF is used in various application areas and represents a multidimensional data analysis. NTF may be an approach to discover hidden patterns in a more holistic, possibly more realistic way (46). In this study, we applied semi-NTF, to study differences from the median level at the baseline of each visit. This approach has two advantages: firstly, the analysis does not have to be limited to non-negative parameters. Secondly, the loss function used by the NTF algorithm tends to disregard values close to zero during the estimation process. In contrast, considering the deviation from the median level provides equal importance to both small and large values of each parameter. The procedure for applying semi-NTF is similar to the procedure described for semi-non-negative matrix factorization (47). The biggest strength of the NTF over the non-parametric test is its ability to discover common patterns of multiple nutrients over multiple time points. On the other hand, it will not specifically search for associations between those patterns with the mode of delivery or provide a significance level. To our knowledge, NTF has not been applied to analyze the nutrient composition of HM and the potential effect the mode of delivery may have on it.

Limitations of this study include the short duration of 4 months and that no infant-related health outcomes were assessed; hence it is difficult to translate the differences of the mode of delivery on HM nutrients to clinically relevant parameters. Also, data regarding maternal diet and antibiotic use were not collected, while both are known to influence the nutrient composition of HM. Further, the reasons why some of the differences occur at specific time points remain unknown. Our study did not differentiate between elective and emergency C-section, which has been shown to have an impact on HM nutrients. Lastly, these preliminary analyses need validation in other larger cohorts.

In summary, our results indicate that using unadjusted analyses, the mode of delivery impacts the HM nutrient composition. HM of mothers who delivered vaginally showed significantly higher levels of n-3 PUFA (anti-inflammatory effects), stearic and palmitoleic acids, and MUFA, whereas HM from mothers undergoing C-section exhibited high levels of n-6 PUFA, as well as higher ARA/DHA and n-6/n-3 ratios (pro-inflammatory effects). We did not observe the differences while adjusting for co-variates, which may suggest that several other factors such as those that were adjusted for in our mixed models (e.g., infant birthweight, parity, gestational age, and the mother’s country which could also be a proxy for her dietary habits) may influence the HM composition. Therefore, future studies should look at human milk as a biological system and study it as an intersection point where maternal biology, diet, environment, infections, and infant factors may have combined effects. Furthermore, our statistical approach allowed visualization of longitudinal patterns that revealed differences in HM nutrient composition between groups of high- and low-density C-section. To our knowledge, this is the first study that examined the effects of mode of delivery on HM components using data from a multicenter European longitudinal cohort. As such it provides a base for future studies on the role of mode of delivery on nutrient composition of HM.

## Data Availability Statement

The datasets presented in this article are not readily available because further sharing of the data was not part of the original subject informed consent. Requests to access the datasets should be directed to corresponding author.

## Ethics Statement

The studies involving human participants were reviewed and approved by Institutional Ethics Board, CHU Bordeaux. The patients/participants provided their written informed consent to participate in this study.

## Author Contributions

TS and ST designed research. TS, FT, FG, SD, and ST wrote the manuscript. LL, CC, and PF conducted the statistical analyses, CM-C, KH, GM, MA, TR, MC, J-CP, and CB conducted the study. ST had primary responsibility for final content. All authors read and approved the final manuscript.

## Conflict of Interest

’This study received funding from Société des Produits Nestlé S.A. The funder had the following involvement with the study: study design, data analysis, interpretation of the results, and reporting in scientific journal. TS, LL, CC, FG, SD, and ST are currently employees of Société des Produits Nestlé S.A. PF and FT are consultants to Société des Produits Nestlé S.A. CM-C, KH, GM, MA, TR, MC, J-CP, and CB received funding from Société des Produits Nestlé S.A. to conduct the study. FT received funding for the research and drafting the manuscript.

## Publisher’s Note

All claims expressed in this article are solely those of the authors and do not necessarily represent those of their affiliated organizations, or those of the publisher, the editors and the reviewers. Any product that may be evaluated in this article, or claim that may be made by its manufacturer, is not guaranteed or endorsed by the publisher.

## Footnotes

1 Log refers to the natural logarithm.

## Supplementary Material

Supplementary Figure 1 Daily infant’s HM intake. Boxplots of infant daily human milk intake (g/day) by delivery mode and by visit. P-values correspond to (two-sided) Mann-Whitney U-tests performed to compare the daily human milk intake between delivery modes at each visit.

## Abbreviations

HM: human milk
C-section: cesarean section
NTF: non-Negative Tensor Factorization
ARA: arachidonic acid
DHA: docosahexaenoic acid
FSLT2: fucosyl-sialyl-lacto-N-tetraose 2
HMO: human milk oligosaccharides
GD3: ganglioside-3
BMI: body mass index
V: visit
MUFA: monousaturated fatty acids
PUFA: polyunsaturated fatty acids.
