# Supplementary material for: Mode of Neonatal Delivery Influences the Nutrient Composition of Human Milk: Results From a Multicenter European Cohort of Lactating Women
Source: Front Nutr. 2022 Apr 6;9:834394. doi: 10.3389/fnut.2022.834394 (PMC9033294; doi:10.3389/fnut.2022.834394)
Supplement: Supplementary file 1 [file Table_1.DOCX]

Supplementary Table 1 Summary of milk components by visit and mode of delivery

|  |  | C-section (Tot. = 80) | | | | | Vaginal (Tot. = 237) | | | | |
| --- | --- | --- | --- | --- | --- | --- | --- | --- | --- | --- | --- |
|  |  | n | Mean | SD | Median | [Q1, Q3] | n | Mean | SD | Median | [Q1, Q3] |
| Fat (g/100ml) |  |  |  |  |  |  |  |  |  |  |  |
|  | V1 | 67 | 2.0 | 1.1 | 1.8 | [1.3, 2.7] | 184 | 1.9 | 1.1 | 1.8 | [1.0, 2.6] |
|  | V2 | 70 | 3.7 | 1.2 | 3.6 | [2.8, 4.4] | 215 | 3.9 | 1.4 | 3.8 | [2.9, 5.0] |
|  | V3 | 63 | 3.6 | 1.6 | 3.5 | [2.3, 4.7] | 194 | 4.0 | 1.5 | 4.0 | [3.0, 5.0] |
|  | V4 | 55 | 4.1 | 1.6 | 4.2 | [3.3, 5.2] | 183 | 4.0 | 1.6 | 4.0 | [2.9, 5.0] |
|  | V5 | 54 | 3.6 | 1.4 | 3.4 | [2.3, 4.4] | 175 | 4.0 | 1.8 | 4.0 | [2.6, 5.4] |
|  | V6 | 55 | 3.8 | 1.9 | 3.6 | [2.5, 4.8] | 165 | 4.1 | 1.8 | 4.1 | [2.7, 5.2] |
| Total protein (g/100ml) |  |  |  |  |  |  |  |  |  |  |  |
|  | V1 | 67 | 2.5 | 1.1 | 2.2 | [1.9, 2.7] | 184 | 2.7 | 1.1 | 2.2 | [1.9, 2.9] |
|  | V2 | 70 | 1.7 | 0.4 | 1.6 | [1.4, 1.8] | 215 | 1.6 | 0.4 | 1.6 | [1.4, 1.8] |
|  | V3 | 63 | 1.5 | 0.4 | 1.4 | [1.3, 1.6] | 194 | 1.5 | 0.3 | 1.5 | [1.3, 1.6] |
|  | V4 | 55 | 1.3 | 0.3 | 1.3 | [1.2, 1.5] | 182 | 1.3 | 0.3 | 1.3 | [1.2, 1.5] |
|  | V5 | 54 | 1.3 | 0.2 | 1.2 | [1.1, 1.4] | 174 | 1.3 | 0.3 | 1.3 | [1.1, 1.4] |
|  | V6 | 55 | 1.3 | 0.3 | 1.2 | [1.1, 1.4] | 165 | 1.3 | 0.5 | 1.2 | [1.1, 1.4] |
| Lactose (g/100ml) |  |  |  |  |  |  |  |  |  |  |  |
|  | V1 | 67 | 5.6 | 1.5 | 5.8 | [5.0, 6.7] | 184 | 5.7 | 1.4 | 5.8 | [5.2, 6.6] |
|  | V2 | 70 | 6.9 | 1.3 | 7.3 | [7.0, 7.5] | 215 | 7.1 | 0.8 | 7.2 | [7.0, 7.4] |
|  | V3 | 63 | 7.2 | 0.8 | 7.4 | [7.1, 7.6] | 194 | 7.1 | 0.7 | 7.3 | [7.0, 7.5] |
|  | V4 | 55 | 7.2 | 0.5 | 7.3 | [7.1, 7.5] | 183 | 7.2 | 0.5 | 7.3 | [7.1, 7.4] |
|  | V5 | 54 | 7.1 | 0.6 | 7.3 | [7.1, 7.5] | 175 | 7.2 | 0.4 | 7.3 | [7.1, 7.5] |
|  | V6 | 55 | 7.2 | 0.7 | 7.3 | [7.1, 7.5] | 165 | 7.1 | 0.7 | 7.3 | [7.0, 7.5] |
| Total PUFA (g/100ml) |  |  |  |  |  |  |  |  |  |  |  |
|  | V1 | 66 | 0.4 | 0.2 | 0.3 | [0.2, 0.5] | 197 | 0.3 | 0.2 | 0.2 | [0.2, 0.4] |
|  | V2 | 70 | 0.5 | 0.3 | 0.5 | [0.3, 0.7] | 215 | 0.5 | 0.2 | 0.4 | [0.3, 0.6] |
|  | V3 | 63 | 0.5 | 0.3 | 0.5 | [0.3, 0.7] | 193 | 0.5 | 0.3 | 0.4 | [0.3, 0.6] |
|  | V4 | 55 | 0.6 | 0.4 | 0.5 | [0.4, 0.7] | 181 | 0.5 | 0.3 | 0.4 | [0.3, 0.6] |
|  | V5 | 54 | 0.5 | 0.3 | 0.5 | [0.3, 0.8] | 173 | 0.5 | 0.3 | 0.4 | [0.3, 0.6] |
|  | V6 | 54 | 0.5 | 0.4 | 0.5 | [0.3, 0.6] | 164 | 0.5 | 0.3 | 0.4 | [0.3, 0.6] |
| PUFA % (%) |  |  |  |  |  |  |  |  |  |  |  |
|  | V1 | 66 | 18.3 | 3.4 | 18.5 | [16.3, 21.3] | 197 | 15.7 | 3.2 | 15.3 | [13.2, 17.7] |
|  | V2 | 70 | 18.1 | 4.5 | 17.9 | [14.9, 21.8] | 215 | 15.5 | 4.2 | 14.7 | [12.4, 18.2] |
|  | V3 | 63 | 18.7 | 5.2 | 18.2 | [15.3, 21.5] | 193 | 15.5 | 4.4 | 14.4 | [12.4, 18.4] |
|  | V4 | 55 | 19.4 | 6.4 | 18.9 | [14.7, 22.5] | 181 | 15.6 | 4.4 | 14.8 | [12.2, 17.8] |
|  | V5 | 54 | 18.5 | 5.9 | 17.2 | [14.2, 21.9] | 173 | 15.6 | 4.9 | 14.0 | [12.0, 18.3] |
|  | V6 | 54 | 18.9 | 6.5 | 17.8 | [13.9, 23.3] | 164 | 15.6 | 4.3 | 14.6 | [12.3, 18.3] |
| MUFA % (%) |  |  |  |  |  |  |  |  |  |  |  |
|  | V1 | 66 | 42.7 | 3.8 | 42.3 | [40.5, 44.9] | 197 | 43.4 | 3.3 | 43.6 | [41.3, 45.4] |
|  | V2 | 70 | 37.8 | 4.6 | 37.7 | [35.2, 40.5] | 215 | 41.6 | 4.2 | 41.4 | [39.1, 44.3] |
|  | V3 | 63 | 39.7 | 4.7 | 39.3 | [36.2, 42.5] | 193 | 42.7 | 4.3 | 42.9 | [40.2, 45.4] |
|  | V4 | 55 | 39.7 | 4.6 | 39.1 | [36.1, 42.2] | 181 | 43.3 | 4.1 | 43.5 | [40.5, 45.9] |
|  | V5 | 54 | 40.0 | 4.6 | 39.5 | [36.9, 42.2] | 173 | 42.4 | 4.8 | 42.7 | [38.9, 45.6] |
|  | V6 | 54 | 38.6 | 4.5 | 38.1 | [34.9, 41.9] | 164 | 42.3 | 4.1 | 42.7 | [39.6, 45.3] |
| SFA % (%) |  |  |  |  |  |  |  |  |  |  |  |
|  | V1 | 66 | 39.0 | 3.8 | 38.3 | [36.6, 40.7] | 197 | 40.9 | 3.9 | 40.6 | [38.2, 43.2] |
|  | V2 | 70 | 44.0 | 5.1 | 44.0 | [40.2, 48.0] | 215 | 42.7 | 5.3 | 42.7 | [38.8, 46.2] |
|  | V3 | 63 | 41.5 | 5.3 | 41.9 | [37.9, 44.7] | 193 | 41.5 | 5.5 | 41.2 | [37.7, 45.3] |
|  | V4 | 55 | 40.8 | 5.8 | 40.4 | [37.4, 44.7] | 181 | 40.9 | 5.2 | 40.9 | [37.3, 43.8] |
|  | V5 | 54 | 41.4 | 5.7 | 42.6 | [37.8, 45.4] | 173 | 41.8 | 5.2 | 41.4 | [38.3, 44.8] |
|  | V6 | 54 | 42.4 | 6.1 | 41.5 | [37.9, 47.4] | 164 | 41.9 | 4.9 | 42.1 | [39.1, 45.0] |
| N-6 to N-3 ratio |  |  |  |  |  |  |  |  |  |  |  |
|  | V1 | 66 | 16.7 | 9.0 | 14.2 | [10.2, 22.5] | 197 | 10.0 | 5.5 | 8.0 | [6.2, 13.3] |
|  | V2 | 70 | 17.3 | 9.0 | 15.6 | [11.1, 21.7] | 215 | 11.5 | 7.1 | 9.1 | [6.2, 14.9] |
|  | V3 | 63 | 19.1 | 11.6 | 14.9 | [10.8, 25.9] | 193 | 12.1 | 7.2 | 9.9 | [6.7, 15.9] |
|  | V4 | 55 | 19.8 | 14.8 | 15.6 | [9.9, 24.3] | 181 | 12.2 | 6.9 | 10.3 | [6.8, 15.2] |
|  | V5 | 54 | 18.7 | 10.8 | 17.5 | [11.6, 22.3] | 173 | 12.6 | 7.4 | 10.0 | [7.3, 15.2] |
|  | V6 | 54 | 18.7 | 10.3 | 16.8 | [11.5, 27.2] | 164 | 12.5 | 7.6 | 10.4 | [7.1, 15.2] |
| ARA to DHA ratio |  |  |  |  |  |  |  |  |  |  |  |
|  | V1 | 66 | 1.9 | 1.0 | 1.7 | [1.2, 2.4] | 197 | 1.4 | 0.6 | 1.2 | [1.0, 1.6] |
|  | V2 | 70 | 1.8 | 1.1 | 1.5 | [1.1, 2.3] | 215 | 1.4 | 0.6 | 1.3 | [1.0, 1.6] |
|  | V3 | 63 | 1.7 | 0.8 | 1.5 | [1.2, 2.0] | 193 | 1.4 | 0.7 | 1.3 | [1.0, 1.7] |
|  | V4 | 55 | 1.9 | 0.8 | 1.8 | [1.3, 2.4] | 181 | 1.5 | 0.7 | 1.4 | [1.0, 1.9] |
|  | V5 | 54 | 1.8 | 0.8 | 1.7 | [1.2, 2.3] | 173 | 1.5 | 0.8 | 1.4 | [1.0, 1.9] |
|  | V6 | 54 | 1.9 | 1.0 | 1.6 | [1.2, 2.4] | 164 | 1.6 | 0.8 | 1.5 | [1.0, 1.9] |
| 16:1 n-7 (mg/100 ml) |  |  |  |  |  |  |  |  |  |  |  |
|  | V1 | 66 | 42.7 | 25.1 | 35.9 | [26.2, 58.5] | 197 | 38.4 | 24.8 | 34.3 | [20.0, 52.3] |
|  | V2 | 70 | 66.1 | 30.6 | 61.9 | [42.6, 86.3] | 215 | 72.3 | 38.0 | 63.8 | [45.8, 87.0] |
|  | V3 | 63 | 68.4 | 42.8 | 55.7 | [41.3, 85.5] | 193 | 79.2 | 38.2 | 73.8 | [49.6, 103.6] |
|  | V4 | 55 | 67.9 | 40.3 | 63.1 | [40.6, 80.1] | 181 | 73.9 | 39.6 | 66.7 | [44.0, 94.5] |
|  | V5 | 54 | 63.6 | 38.9 | 56.5 | [38.9, 73.3] | 173 | 69.0 | 42.6 | 62.4 | [39.8, 90.3] |
|  | V6 | 54 | 61.2 | 46.1 | 46.5 | [29.3, 84.1] | 164 | 69.1 | 40.0 | 63.0 | [41.5, 91.3] |
| 18:0 (mg/100 ml) |  |  |  |  |  |  |  |  |  |  |  |
|  | V1 | 66 | 109.3 | 52.0 | 100.1 | [72.2, 139.8] | 197 | 119.5 | 69.4 | 103.1 | [67.2, 154.8] |
|  | V2 | 70 | 173.1 | 81.4 | 166.6 | [119.9, 206.6] | 215 | 190.7 | 93.0 | 181.1 | [122.4, 240.8] |
|  | V3 | 63 | 175.1 | 102.6 | 165.6 | [111.4, 204.2] | 193 | 203.9 | 90.6 | 200.1 | [136.0, 258.8] |
|  | V4 | 55 | 193.0 | 90.4 | 182.5 | [125.9, 249.0] | 181 | 209.5 | 107.4 | 193.4 | [134.0, 264.1] |
|  | V5 | 54 | 167.2 | 83.6 | 151.9 | [112.5, 214.5] | 173 | 203.7 | 112.8 | 196.4 | [120.1, 252.3] |
|  | V6 | 54 | 174.4 | 105.2 | 154.7 | [101.8, 221.7] | 164 | 210.6 | 117.5 | 198.1 | [127.1, 272.8] |
| 18:1 n-9 (mg/100 ml) |  |  |  |  |  |  |  |  |  |  |  |
|  | V1 | 66 | 686.6 | 318.4 | 615.8 | [500.4, 867.1] | 197 | 681.1 | 363.7 | 610.8 | [394.1, 899.4] |
|  | V2 | 70 | 940.8 | 388.6 | 890.3 | [661.6, 1139.7] | 215 | 1062.2 | 473.6 | 1001.8 | [719.6, 1341.5] |
|  | V3 | 63 | 992.5 | 532.5 | 914.3 | [625.9, 1195.7] | 193 | 1144.9 | 477.5 | 1101.5 | [799.3, 1412.5] |
|  | V4 | 55 | 1060.1 | 498.5 | 1071.8 | [682.8, 1402.9] | 181 | 1164.4 | 584.3 | 1051.4 | [725.9, 1488.0] |
|  | V5 | 54 | 976.6 | 483.5 | 948.6 | [568.1, 1240.2] | 173 | 1114.0 | 607.9 | 1061.5 | [693.8, 1448.3] |
|  | V6 | 54 | 955.5 | 561.9 | 874.6 | [581.9, 1171.7] | 164 | 1130.3 | 593.7 | 1102.9 | [692.8, 1487.3] |
| 18:2 n-6 (mg/100 ml) |  |  |  |  |  |  |  |  |  |  |  |
|  | V1 | 66 | 283.7 | 164.6 | 252.6 | [168.7, 340.6] | 197 | 219.4 | 137.3 | 184.1 | [122.2, 279.9] |
|  | V2 | 70 | 447.6 | 232.6 | 390.4 | [265.9, 603.3] | 215 | 370.9 | 192.5 | 339.2 | [229.3, 449.9] |
|  | V3 | 63 | 462.8 | 294.6 | 417.1 | [268.8, 561.5] | 193 | 405.8 | 237.2 | 342.1 | [253.1, 487.8] |
|  | V4 | 55 | 532.1 | 386.5 | 416.9 | [319.4, 636.7] | 181 | 404.5 | 242.4 | 336.1 | [234.5, 526.5] |
|  | V5 | 54 | 455.6 | 272.5 | 388.6 | [274.4, 652.5] | 173 | 408.0 | 296.8 | 322.1 | [210.2, 527.5] |
|  | V6 | 54 | 470.0 | 344.0 | 422.7 | [249.9, 555.3] | 164 | 400.3 | 242.4 | 357.7 | [214.7, 539.7] |
| 18:3 n-3 (mg/100 ml) |  |  |  |  |  |  |  |  |  |  |  |
|  | V1 | 66 | 11.9 | 7.8 | 10.6 | [6.2, 15.2] | 197 | 16.6 | 12.3 | 13.6 | [7.6, 22.9] |
|  | V2 | 70 | 20.1 | 14.2 | 15.7 | [10.4, 25.8] | 215 | 28.4 | 23.0 | 21.1 | [12.7, 37.2] |
|  | V3 | 63 | 20.9 | 16.4 | 15.5 | [9.7, 24.9] | 193 | 30.2 | 23.5 | 22.7 | [15.1, 37.4] |
|  | V4 | 55 | 24.9 | 21.0 | 19.1 | [12.6, 28.5] | 181 | 33.0 | 33.6 | 21.7 | [13.7, 35.7] |
|  | V5 | 54 | 20.2 | 15.0 | 15.1 | [9.8, 24.8] | 173 | 29.4 | 26.8 | 23.1 | [12.5, 36.8] |
|  | V6 | 54 | 22.8 | 20.3 | 15.1 | [9.8, 26.4] | 164 | 31.0 | 28.6 | 23.6 | [13.7, 37.9] |
| 20:0 (mg/100 ml) |  |  |  |  |  |  |  |  |  |  |  |
|  | V1 | 66 | 3.7 | 1.8 | 3.3 | [2.6, 5.0] | 197 | 4.4 | 2.4 | 4.0 | [2.7, 5.6] |
|  | V2 | 70 | 5.1 | 2.7 | 4.7 | [3.5, 6.1] | 215 | 5.5 | 2.7 | 5.4 | [3.7, 6.9] |
|  | V3 | 63 | 4.9 | 2.8 | 4.5 | [3.0, 5.9] | 193 | 5.5 | 2.5 | 5.3 | [3.9, 6.9] |
|  | V4 | 55 | 5.2 | 2.6 | 5.1 | [3.3, 6.4] | 181 | 5.5 | 3.0 | 5.1 | [3.6, 7.0] |
|  | V5 | 54 | 4.4 | 2.4 | 4.0 | [2.7, 5.7] | 173 | 5.3 | 3.0 | 5.2 | [3.3, 6.6] |
|  | V6 | 54 | 4.5 | 3.1 | 4.1 | [2.5, 5.7] | 164 | 5.4 | 3.1 | 5.0 | [3.2, 7.2] |
| 20:2 n-6 (mg/100 ml) |  |  |  |  |  |  |  |  |  |  |  |
|  | V1 | 66 | 17.2 | 9.5 | 16.1 | [10.1, 21.3] | 197 | 14.2 | 8.7 | 12.2 | [8.1, 18.1] |
|  | V2 | 70 | 13.4 | 6.1 | 12.3 | [8.8, 17.7] | 215 | 10.8 | 5.6 | 10.0 | [6.8, 13.0] |
|  | V3 | 63 | 11.5 | 6.4 | 9.9 | [7.0, 14.5] | 193 | 10.0 | 5.3 | 8.8 | [6.4, 12.1] |
|  | V4 | 55 | 10.4 | 5.6 | 9.5 | [6.4, 13.1] | 181 | 8.4 | 4.9 | 7.0 | [4.9, 10.6] |
|  | V5 | 54 | 9.0 | 5.4 | 7.4 | [4.8, 12.7] | 173 | 7.7 | 5.1 | 6.8 | [4.2, 10.6] |
|  | V6 | 54 | 8.5 | 5.0 | 8.0 | [5.3, 10.6] | 164 | 7.7 | 4.6 | 7.0 | [4.3, 10.3] |
| 20:5 n-3 (mg/100 ml) |  |  |  |  |  |  |  |  |  |  |  |
|  | V1 | 66 | 1.4 | 1.3 | 1.0 | [1.0, 1.0] | 197 | 1.3 | 0.9 | 1.0 | [1.0, 1.0] |
|  | V2 | 70 | 2.1 | 1.8 | 1.0 | [1.0, 2.6] | 215 | 2.5 | 2.2 | 1.0 | [1.0, 3.2] |
|  | V3 | 63 | 1.8 | 1.3 | 1.0 | [1.0, 2.4] | 192 | 2.6 | 2.6 | 2.0 | [1.0, 3.2] |
|  | V4 | 55 | 2.1 | 2.2 | 1.0 | [1.0, 2.6] | 181 | 2.5 | 2.5 | 1.0 | [1.0, 3.0] |
|  | V5 | 54 | 1.8 | 1.8 | 1.0 | [1.0, 2.1] | 173 | 2.5 | 2.5 | 1.0 | [1.0, 3.0] |
|  | V6 | 54 | 1.9 | 1.6 | 1.0 | [1.0, 2.3] | 163 | 2.3 | 2.3 | 1.0 | [1.0, 2.8] |
| 22:1 n-9 (mg/100 ml) |  |  |  |  |  |  |  |  |  |  |  |
|  | V1 | 66 | 3.7 | 2.0 | 3.3 | [2.6, 4.6] | 197 | 4.1 | 2.3 | 3.7 | [2.5, 5.1] |
|  | V2 | 70 | 2.5 | 1.4 | 2.4 | [1.0, 3.1] | 215 | 2.8 | 1.7 | 2.6 | [1.0, 3.6] |
|  | V3 | 63 | 2.1 | 1.7 | 1.0 | [1.0, 2.8] | 193 | 2.6 | 1.6 | 2.5 | [1.0, 3.2] |
|  | V4 | 55 | 1.8 | 1.1 | 1.0 | [1.0, 2.3] | 181 | 2.2 | 1.7 | 1.0 | [1.0, 2.8] |
|  | V5 | 54 | 1.6 | 1.2 | 1.0 | [1.0, 2.3] | 173 | 2.0 | 1.4 | 1.0 | [1.0, 2.7] |
|  | V6 | 54 | 1.6 | 1.1 | 1.0 | [1.0, 1.8] | 164 | 2.1 | 1.5 | 1.0 | [1.0, 2.7] |
| 22:6 n-3 (mg/100 ml) |  |  |  |  |  |  |  |  |  |  |  |
|  | V1 | 66 | 11.5 | 9.2 | 8.3 | [5.5, 14.5] | 197 | 13.2 | 8.4 | 11.0 | [7.4, 16.7] |
|  | V2 | 70 | 12.6 | 7.8 | 10.4 | [7.3, 16.1] | 215 | 14.7 | 10.5 | 12.5 | [8.0, 17.7] |
|  | V3 | 63 | 10.1 | 5.6 | 9.1 | [5.2, 13.8] | 193 | 13.3 | 10.5 | 11.2 | [7.0, 15.4] |
|  | V4 | 55 | 9.9 | 6.5 | 8.5 | [5.2, 11.9] | 181 | 11.0 | 8.2 | 9.0 | [6.1, 13.1] |
|  | V5 | 54 | 8.7 | 5.6 | 8.0 | [4.3, 10.9] | 173 | 10.4 | 8.8 | 8.6 | [5.2, 13.2] |
|  | V6 | 54 | 8.5 | 6.0 | 7.6 | [4.5, 10.0] | 163 | 9.7 | 7.0 | 7.9 | [4.8, 12.6] |
| Calcium (mg/l) |  |  |  |  |  |  |  |  |  |  |  |
|  | V1 | 55 | 268.9 | 57.2 | 276.8 | [235.1, 312.7] | 136 | 280.0 | 78.6 | 276.0 | [222.8, 319.8] |
|  | V2 | 68 | 278.8 | 52.8 | 279.4 | [262.0, 305.9] | 209 | 293.0 | 55.3 | 290.2 | [260.2, 322.1] |
|  | V3 | 60 | 289.0 | 46.9 | 294.6 | [254.0, 309.8] | 189 | 297.6 | 48.8 | 300.3 | [259.5, 329.0] |
|  | V4 | 54 | 289.8 | 42.6 | 285.8 | [257.9, 312.0] | 180 | 303.8 | 42.6 | 302.4 | [276.2, 328.0] |
|  | V5 | 52 | 280.8 | 42.5 | 278.7 | [248.3, 301.8] | 171 | 298.5 | 46.3 | 294.1 | [265.9, 320.6] |
|  | V6 | 53 | 270.2 | 39.1 | 266.9 | [242.0, 284.0] | 159 | 283.3 | 39.4 | 282.0 | [258.1, 305.5] |
| Phosphorus (mg/l) |  |  |  |  |  |  |  |  |  |  |  |
|  | V1 | 55 | 119.6 | 37.5 | 113.1 | [93.9, 142.1] | 136 | 131.6 | 40.7 | 127.4 | [103.2, 156.4] |
|  | V2 | 68 | 159.1 | 29.3 | 160.7 | [142.2, 172.8] | 209 | 164.2 | 30.3 | 162.0 | [147.2, 183.5] |
|  | V3 | 60 | 149.9 | 26.5 | 148.3 | [134.7, 168.2] | 189 | 153.9 | 24.4 | 153.4 | [138.7, 167.9] |
|  | V4 | 54 | 137.1 | 22.4 | 135.5 | [120.7, 148.4] | 180 | 139.5 | 20.5 | 140.2 | [124.8, 152.2] |
|  | V5 | 52 | 126.1 | 17.1 | 125.2 | [114.1, 134.8] | 171 | 132.6 | 20.5 | 132.2 | [122.3, 144.9] |
|  | V6 | 53 | 129.4 | 21.8 | 124.0 | [114.3, 141.7] | 159 | 129.3 | 21.1 | 128.7 | [114.9, 141.6] |
| Zinc (µg/l) |  |  |  |  |  |  |  |  |  |  |  |
|  | V1 | 55 | 6417.1 | 2561.7 | 6168.6 | [4486.7, 8347.2] | 136 | 8047.8 | 2807.6 | 7827.9 | [6114.7, 9859.4] |
|  | V2 | 68 | 3411.3 | 1226.6 | 3408.1 | [2743.8, 4307.7] | 209 | 3507.9 | 1085.5 | 3478.1 | [2833.8, 4232.7] |
|  | V3 | 60 | 2597.4 | 897.2 | 2747.1 | [1982.2, 3195.1] | 189 | 2582.2 | 863.3 | 2547.8 | [1988.6, 3161.4] |
|  | V4 | 54 | 1478.0 | 582.5 | 1457.6 | [1035.4, 1938.6] | 180 | 1597.3 | 744.5 | 1600.1 | [1028.9, 2027.4] |
|  | V5 | 52 | 1129.7 | 551.7 | 996.3 | [785.6, 1329.7] | 168 | 1186.5 | 548.5 | 1098.4 | [748.4, 1536.5] |
|  | V6 | 53 | 1052.6 | 596.9 | 961.2 | [682.9, 1283.3] | 159 | 985.9 | 468.7 | 858.0 | [643.6, 1232.3] |
| Manganese (µg/l) |  |  |  |  |  |  |  |  |  |  |  |
|  | V1 | 45 | 5.0 | 2.8 | 4.0 | [3.0, 6.3] | 118 | 6.7 | 3.7 | 5.5 | [4.1, 8.2] |
|  | V2 | 31 | 4.0 | 1.4 | 3.4 | [3.0, 4.4] | 108 | 4.1 | 1.3 | 3.7 | [3.2, 4.6] |
|  | V3 | 20 | 4.1 | 1.3 | 4.0 | [2.9, 4.8] | 77 | 3.5 | 1.0 | 3.2 | [2.8, 3.9] |
|  | V4 | 10 | 5.1 | 4.4 | 3.6 | [3.1, 4.5] | 56 | 3.6 | 1.4 | 3.1 | [2.7, 3.9] |
|  | V5 | 11 | 3.6 | 1.1 | 3.3 | [3.1, 3.7] | 43 | 3.3 | 0.7 | 3.1 | [2.8, 3.5] |
|  | V6 | 12 | 3.8 | 0.7 | 3.9 | [3.4, 4.3] | 36 | 4.0 | 2.3 | 3.4 | [3.1, 4.0] |
| Iodine (µg/l) |  |  |  |  |  |  |  |  |  |  |  |
|  | V1 | 53 | 341.4 | 249.5 | 288.0 | [143.6, 533.2] | 131 | 179.9 | 196.1 | 120.7 | [58.7, 196.8] |
|  | V2 | 69 | 168.4 | 99.3 | 148.6 | [103.1, 201.6] | 213 | 144.5 | 95.1 | 122.9 | [83.3, 183.3] |
|  | V3 | 62 | 116.9 | 58.9 | 105.1 | [70.9, 155.7] | 191 | 119.6 | 85.0 | 93.8 | [69.4, 146.4] |
|  | V4 | 55 | 101.2 | 68.2 | 86.7 | [53.9, 122.1] | 181 | 95.2 | 56.6 | 82.0 | [55.5, 116.7] |
|  | V5 | 53 | 93.5 | 55.7 | 76.1 | [55.0, 117.1] | 173 | 92.8 | 57.2 | 76.8 | [56.6, 112.1] |
|  | V6 | 54 | 91.8 | 58.3 | 68.4 | [57.2, 100.9] | 160 | 93.6 | 91.8 | 71.9 | [47.9, 111.3] |
| Selenium (µg/l) |  |  |  |  |  |  |  |  |  |  |  |
|  | V1 | 55 | 29.0 | 13.1 | 25.5 | [21.5, 32.7] | 134 | 30.6 | 13.7 | 26.9 | [22.7, 33.8] |
|  | V2 | 68 | 19.3 | 5.8 | 18.5 | [16.8, 20.5] | 209 | 17.8 | 3.8 | 17.3 | [15.2, 19.7] |
|  | V3 | 60 | 15.7 | 2.5 | 15.9 | [14.0, 17.2] | 189 | 15.2 | 2.9 | 15.0 | [13.1, 16.9] |
|  | V4 | 54 | 12.3 | 2.5 | 11.9 | [10.6, 13.5] | 180 | 11.9 | 2.6 | 11.5 | [10.3, 13.3] |
|  | V5 | 52 | 10.7 | 2.0 | 10.4 | [9.3, 11.8] | 171 | 10.4 | 1.9 | 10.3 | [9.0, 11.4] |
|  | V6 | 53 | 10.1 | 2.2 | 9.7 | [8.5, 11.6] | 159 | 9.8 | 5.9 | 9.2 | [8.2, 10.3] |
| GD3 (ug/ml) |  |  |  |  |  |  |  |  |  |  |  |
|  | V1 | 62 | 6.9 | 3.5 | 6.4 | [4.3, 8.9] | 178 | 8.2 | 4.2 | 7.7 | [5.5, 10.6] |
|  | V2 | 69 | 4.2 | 3.3 | 3.5 | [2.1, 4.8] | 215 | 3.8 | 2.8 | 3.2 | [2.2, 4.5] |
|  | V3 | 63 | 2.8 | 2.3 | 2.4 | [1.5, 3.4] | 194 | 2.7 | 1.6 | 2.3 | [1.4, 3.5] |
|  | V4 | 55 | 2.3 | 2.0 | 1.5 | [1.0, 3.2] | 183 | 2.3 | 2.1 | 1.7 | [1.0, 2.8] |
|  | V5 | 53 | 2.3 | 2.2 | 1.6 | [0.7, 3.1] | 175 | 1.8 | 1.7 | 1.3 | [0.7, 2.6] |
|  | V6 | 54 | 2.4 | 3.0 | 1.2 | [0.5, 3.2] | 165 | 1.9 | 2.2 | 1.2 | [0.7, 2.4] |
| Lactoferrin (ng/µl) |  |  |  |  |  |  |  |  |  |  |  |
|  | V1 | 62 | 6104.1 | 3008.4 | 5935.5 | [4476.5, 7494.6] | 163 | 5970.7 | 2701.3 | 5506.5 | [4377.3, 6881.9] |
|  | V2 | 70 | 3212.6 | 2801.4 | 2521.8 | [1933.8, 3467.2] | 214 | 2542.5 | 1125.4 | 2306.9 | [1841.4, 2996.0] |
|  | V3 | 62 | 2423.3 | 2518.5 | 1969.4 | [1552.7, 2453.0] | 193 | 1862.3 | 816.0 | 1689.0 | [1300.4, 2243.2] |
|  | V4 | 55 | 1612.6 | 805.9 | 1428.2 | [1131.0, 1941.1] | 183 | 1430.9 | 668.1 | 1269.3 | [992.6, 1718.9] |
|  | V5 | 53 | 1534.3 | 739.3 | 1375.8 | [1018.2, 1834.2] | 175 | 1326.5 | 800.2 | 1168.5 | [883.6, 1581.2] |
|  | V6 | 53 | 1401.5 | 732.2 | 1233.6 | [970.9, 1487.2] | 163 | 1269.3 | 1157.9 | 1018.2 | [815.3, 1404.4] |

Sample size, mean, standard deviation (SD), median, 1st quartile (Q1 = 25th percentile), 3rd quartile (Q3 = 75th percentile)
