# Supplementary figures and images for: Mode of Neonatal Delivery Influences the Nutrient Composition of Human Milk: Results From a Multicenter European Cohort of Lactating Women
Source: Front Nutr. 2022 Apr 6;9:834394. doi: 10.3389/fnut.2022.834394 (PMC9033294; doi:10.3389/fnut.2022.834394)

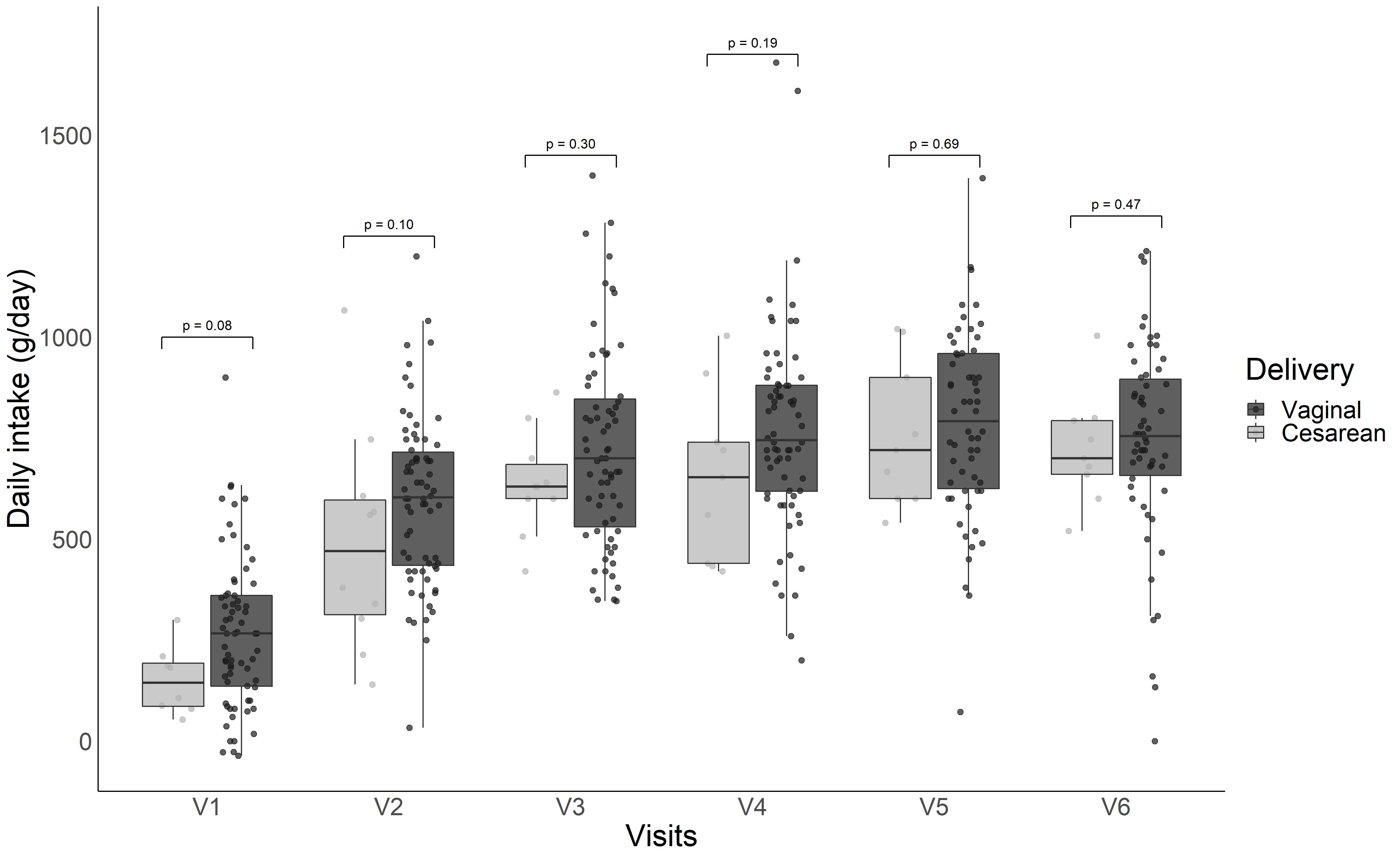

Supplement: Supplementary Figure 1 — Daily infant’s HM intake. Boxplots of infant daily human milk intake (g/day) by delivery mode and by visit. P-values correspond to (two-sided) Mann-Whitney U-tests performed to compare the daily human milk intake between delivery modes at each visit. [file Image_1.JPEG]
